# From guided self-help to comprehensive ED treatment

**DOI:** 10.1192/j.eurpsy.2024.640

**Published:** 2024-08-27

**Authors:** B. Babusa, K. Bai-Nagy, J. Kovács, Á. Dósa, G. Salavecz, F. Túry, G. Purebl

**Affiliations:** ^1^Semmelweis University, Faculty of Medicine, Institute of Behavioural Sciences, Budapest, Hungary

## Abstract

**Introduction:**

The incidence of eating disorders is increasing in Hungary and Central-Eastern Europe. The number of complex/severe cases is also increasing. Accordingly, several new unmet needs of the users and their relatives appear in the clinical care.

**Objectives:**

As a possible response to these unmet needs, we have introduced a multifaceted care model for eating disorders. To facilitate easily accessible yet effective care close to home, a support programme with an online guided self-help tool and regular consultations with first responder psychiatrists or clinical psychologists has been introduced. For non (or partial) responders, a multi-faceted modular treatment programme has been developed with an individualised combination of different therapeutic approaches, including family therapy, dialectical behaviour therapy (DBT) specific to binge eating disorder and bulimia, CBT and the use of virtual reality as an adjunct treatment. The most severe cases are referred for (also multifaceted) inpatient treatment. In terms of research, we want to focus on the key issues for rapid, cost-effective treatment. Firstly, we want to develop an individual profiling system at the start of therapy to assess which individual combination of modules can produce a rapid therapeutic response. Secondly, we want to identify the active gamechanger elements of therapy that are associated with the greatest change in symptoms.

**Methods:**

Patients complete the following questionnaires:

-in the guided self-help group: Eating disoreder inventory, (EDI-I), McMaster Family Assessment Device (FAD), Eastin Disorder Diagnostic Scale (EDDS), Eating Behavioral Severity Scale, Eating Disorders Symptom Impact Scale (EDSIS-S)

- in DBT groups: Eating Disorder Examination Questionnarie (EDE-Q), Three Factor Eating Questionnaire-R21, Rosenberg Self-Esteem Scale, Patient Health Questionnaire-(PHQ-9), Cognitive Emotion Regulation Questionnaire (CERQ)

- in individual therapies: Mini International Neuropsychiatric Interview (MINI) and Structured Clinical Interview for DSM 5- Alternative Model for Personality Disorders (SCIP-5-AMPD), EDI-I., Mentalization Questionnaire (MZQ), Dissociation Questionnaire (DIS-Q), Symptom Checklist-90 (SCL-90), (PHQ-9), Childhood Trauma Questionnaire (CTQ) and Young Parenting Inventory (YPI).

**Results:**

Patient recruitment and therapies are currently underway, the first preliminary results are expected in the spring period.

**Conclusions:**

In order to provide individualized care more effectively, it is important to identify the factors that determine which therapeutic modalities work best for the patient.

**Disclosure of Interest:**

None Declared

